# Efficacy of chlorthalidone and hydrochlorothiazide in combination with amiloride in multiple doses on blood pressure in patients with primary hypertension: a protocol for a factorial randomized controlled trial

**DOI:** 10.1186/s13063-019-3909-z

**Published:** 2019-12-16

**Authors:** Vítor Magnus Martins, Lucas Helal, Filipe Ferrari, Leonardo Grabinski Bottino, Sandra Costa Fuchs, Flávio Danni Fuchs

**Affiliations:** 1 Graduate Program in Cardiology and Cardiovascular Sciences, Hospital de Clínicas de Porto Alegre, Universidade Federal do Rio Grande do Sul, Porto Alegre, RS Brazil; 2 Division of Cardiology, Hospital de Clínicas de Porto Alegre, Porto Alegre, RS Brazil

**Keywords:** Diuretics, Thiazides, Amiloride, Blood pressure, Hypertension, Treatment

## Abstract

**Background:**

Thiazide diuretics have demonstrated favorable blood pressure lowering efficacy, but the equivalent doses of their more common agents, chlorthalidone and hydrochlorothiazide, are still unclear. Further, concerns exist regarding adverse metabolic effects, which may be attenuated with the concomitant administration of a potassium-sparing diuretic, such as amiloride. This trial aims to investigate the efficacy of chlorthalidone and hydrochlorothiazide, in combination with amiloride at different doses, for initial management of patients with primary hypertension.

**Methods/design:**

This is a factorial (2 × 2) randomized double-blinded clinical trial comparing the association of a thiazide diuretic (chlorthalidone 25 mg/day or hydrochlorothiazide 50 mg/day) with a potassium-sparing diuretic (amiloride 10 mg/day or amiloride 20 mg/day) in patients with primary hypertension. The primary outcome will be the mean change from baseline in 24-h systolic and diastolic blood pressure measured by ambulatory blood pressure monitoring. The secondary outcomes will be the mean change from baseline in daytime and nighttime systolic and diastolic blood pressure measured by ambulatory blood pressure monitoring, mean change from baseline in systolic and diastolic blood pressure measured by office blood pressure, incidence of adverse events, variation of laboratory parameters, and proportion of patients who achieved blood pressure control. The follow-up will last 12 weeks. For a P alpha of 0.05, power of 80%, standard deviation of 9 mmHg, and absolute difference of 6 mmHg on systolic blood pressure on 24-h ambulatory blood pressure monitoring, it will be necessary to study a total of 76 patients. The sample size will be increased by 10% to compensate for losses, resulting in 84 patients being randomized.

**Discussion:**

Diuretics are pivotal drugs for the treatment of hypertension. Chlorthalidone and hydrochlorothiazide, in combination with amiloride in multiple doses, will be tested in terms of blood pressure lowering efficacy and safety. Since the intensity of blood pressure reduction is the major determinant of reduction in cardiovascular risk in hypertensive patients, this study will help to determine which combination of diuretics represents the most appropriate treatment for this population.

**Trial registration:**

ClinicalTrials.gov, NCT03928145. Registered on 25 April 2019. Last update on 29 April 2019.

## Background

Thiazide diuretics have been commonly used as pharmacological agents for the treatment of hypertension for over five decades [1, 2], remaining the cornerstone of antihypertensive treatment due to their favorable blood pressure (BP) lowering efficacy, safety profile, and low cost. In patients with primary hypertension, thiazide diuretics have been demonstrated to be effective at low doses [3–10], while higher doses produce more side effects, often with little further reduction in BP [4]. Chlorthalidone (CTD) has been shown to provide greater antihypertensive efficacy than hydrochlorothiazide (HCTZ) at similar dose levels [11–15] with no evidence of higher incidence of side effects [15]. CTD is 1.5 to 2 times as effective as HCTZ at lowering BP at the same dose [12]. The smaller efficacy of HCTZ may be explained by a shorter duration of action compared to CTD [11, 12, 16]. Thus, when compared to HCTZ, these characteristics of CTD could promote better results in reducing BP and cardiovascular outcomes [17].

The use of thiazide diuretics may be associated with adverse metabolic effects, such as hypokalemia, hyponatremia, hyperuricemia, hyperglycemia, hyperlipidemia, and hypomagnesemia [4, 18, 19]. The incidence of these metabolic complications increases in a dose–response manner [3, 4, 20, 21]. It is estimated that less than half of the patients receiving thiazide diuretics develop hypokalemia (serum potassium < 3.5 mEq/L) [22]. The beneficial effect of chlorthalidone in the prevention of major cardiovascular events in the SHEP trial was lost when potassium dropped below 3.5 mEq/L [23]. The risk of hypokalemia may be minimized by combining thiazides with blockers of the epithelial sodium channel (e.g., amiloride and triamterene), which may also mitigate the impaired glucose tolerance associated with thiazides [24]. The blockers of the epithelial sodium channel are commonly referred to as potassium-sparing diuretics. Although the antihypertensive properties of the thiazide diuretics have been well documented [3–10], the BP lowering effect of potassium-sparing diuretics has not been as clearly determined [25]. However, some studies suggest that amiloride may be valuable in treating resistant hypertension [26] and may have a more potent antihypertensive effect in higher doses [24, 27].

It remains unknown whether different diuretics are associated with different clinical outcomes. Both CTD and indapamide have been shown to reduce cardiovascular events in landmark randomized trials [28, 29], whereas there is no evidence that HCTZ alone reduces cardiovascular events [30]. Despite opinions on the preference of CTD and indapamide over classic thiazide diuretics (e.g., HCTZ) [31], no randomized controlled trials have directly compared HCTZ versus CTD in relation to cardiovascular outcomes in hypertensive patients. This scenario supports the relevance of comparative testing of these drugs. Substantial clinical evidence concluded that the amount of BP reduction is the major determinant of reduction in cardiovascular risk in hypertensive patients [21, 32–34], which renders the BP lowering effect among diuretics an appropriate surrogate outcome.

So, we designed a factorial trial to compare the BP-lowering efficacy and safety profile of CTD and HCTZ, in combination with amiloride in multiple doses, in patients with primary hypertension. The mean change from baseline in 24-h systolic and diastolic BP measured by ambulatory blood pressure monitoring (ABPM) is the primary outcome. The mean change from baseline in daytime and nighttime systolic and diastolic BP measured by ABPM, mean change from baseline in systolic and diastolic BP measured by office BP, incidence of adverse events, variation of laboratory parameters, and proportion of patients who achieved BP control are the secondary outcomes.

### Primary objectives

To compare CTD 25 mg with amiloride 20 mg versus other combinations of thiazide with amiloride, with respect to BP-lowering efficacy, in subjects with primary hypertension.

### Secondary objectives

To compare, in subjects with primary hypertension, CTD 25 mg with amiloride 20 mg versus other combinations of thiazide with amiloride, with respect to the incidence of adverse events, variation of laboratory parameters, and proportion of patients who achieved BP control.

## Methods/design

### Study design

This is a randomized double-blind single-center superiority trial, controlled by active treatment, with factorial design (2 × 2), with a 1:1:1:1 allocation ratio, follow-up period of 12 weeks, and a primary endpoint of mean change from baseline in 24-h systolic and diastolic BP measured by ABPM. Eligible participants will be randomized to receive two simultaneous interventions: a thiazide diuretic (CTD 25 mg/day or HCTZ 50 mg/day) and a potassium-sparing diuretic (amiloride 10 mg/day or amiloride 20 mg/day).

### Study setting

The study will be conducted in the Center for Clinical Research of Hospital de Clínicas de Porto Alegre (HCPA), which aims to establish guidelines and policies regarding the conduct of clinical research as a whole. In this sense, it provides adequate infrastructure for the development of all stages of clinical and epidemiological studies, in line with the public health needs of Brazil.

Inclusion criteria:

- Adults (aged 30 to 75 years) with diagnosis of primary hypertension based on ABPM (mean 24-h systolic BP ≥ 130 mmHg or mean 24-h diastolic BP ≥ 80 mmHg)
- No current use of antihypertensive medication
- Written consent for participation in the study

If the patient is on antihypertensive monotherapy prior to randomization and has BP below 160/100 mmHg (as measured by office BP), he may have his medication suspended for 2 weeks to confirm the inclusion criteria (washout phase).

Exclusion criteria:

- Low life expectancy
- Other indications for the use of diuretics
- Intolerance or contraindications to the study drugs
- Cardiovascular disease (heart failure, myocardial infarction or stroke)
- Secondary hypertension
- Chronic kidney disease and/or abnormal renal function (creatinine > 1.5 mg/dL)
- Hyperkalemia (serum potassium > 5.5 mEq/L)
- Gout
- Previous antihypertensive treatment with more than one drug
- Systolic BP ≥ 160 mmHg or diastolic BP ≥ 100 mmHg measured through office BP
- Pregnancy or prospective pregnancy during the study
- Lactating women

### Interventions

The trial has a factorial design, where participants will receive two simultaneous interventions: a thiazide diuretic (CTD 25 mg or HCTZ 50 mg) and a potassium-sparing diuretic (amiloride 10 mg or amiloride 20 mg). Participants will be randomly assigned to four parallel groups:

- CTD 25 mg + amiloride 10 mg
- CTD 25 mg + amiloride 20 mg
- HCTZ 50 mg + amiloride 10 mg
- HCTZ 50 mg + amiloride 20 mg

The thiazide diuretic and amiloride will be combined in a single capsule, which will be provided by a compounding pharmacy. The medication will be administered as fixed-dose combinations. Patients will be instructed to take the medication orally in the morning upon waking. Adherence to trial medication will be assessed by means of pill count.

See Additional file 1 for the Template for Intervention Description and Replication (TIDieR) checklist.

### Outcomes

Primary outcomes:

- Difference between the treatment arms in mean change from baseline in 24-h systolic and diastolic BP measured by ABPM at 12 weeks

Secondary outcomes:

- Difference between the treatment arms in mean change from baseline to 12 weeks in: daytime and nighttime systolic and diastolic BP measured by ABPM; systolic and diastolic BP measured by office BP; laboratory parameters.
- Difference between treatment arms in the proportion of participants reporting adverse events in the 12 weeks following randomization.
- Difference between treatment arms in the proportion of participants achieving BP control at 12 weeks. BP control will be defined as < 140/90 mmHg and < 130/80 mmHg for office BP and 24-h ABPM, respectively.

### Participant timeline

Participants will be recruited from outpatient clinics and from Basic Health Units (public health system) in Porto Alegre, Brazil, and then invited to participate in the study.

The first visit will consist of (1) informed consent signing, (2) eligibility assessment, and (3) sociodemographic and clinic data collection. If the patient is taking an antihypertensive drug and has BP below 160/100 mmHg (as measured by office BP), he will have his medication suspended for 2 weeks to confirm the eligibility criteria (washout phase, which allows most of the effects of the BP drug to vanish). If no antihypertensive medication is being used, BP measurements will be carried out in the office (average of three measurements) and ABPM will be placed. Then, participants will be instructed to return the next day after fasting (12 h) for laboratory tests.

The next visit will consist of removal of ABPM, confirmation of primary hypertension through ABPM (mean 24-h systolic BP ≥ 130 mmHg or mean 24-h diastolic BP ≥ 80 mmHg), office BP measurement (average of three measurements), anthropometric evaluation, and laboratory tests. After confirming the eligibility criteria, randomization and delivery of the study drug will be performed.

The intermediate visit (week 6) will consist of office BP measurement (average of three measurements), evaluation of adherence to treatment, investigation of adverse events, and delivery of study medication. Participants will be instructed to return in 6 weeks after fasting (12 h) for laboratory tests.

The close-out visit (week 12) will consist of office BP measurement (average of three measurements), anthropometric evaluation, evaluation of adherence to treatment, investigation of adverse events, laboratory tests, and placement of ABPM. Participants will be instructed to return the next day for removal of ABPM, verification of laboratory test results, and termination of study participation.

**Fig. 1:**
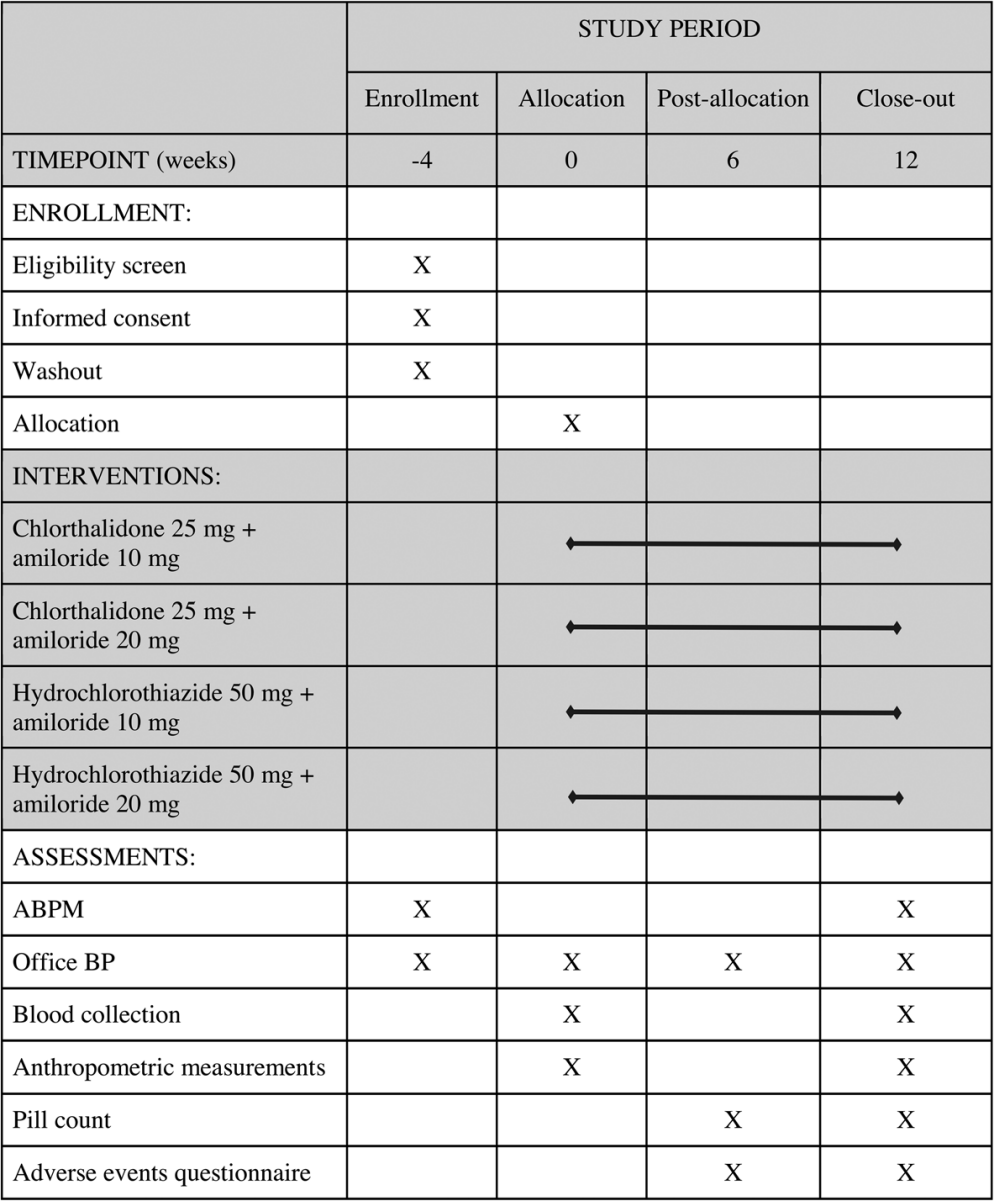
Schedule of enrollment, interventions and assessments. *ABPM* ambulatory blood pressure monitoring, *BP* blood pressure

**Fig. 2:**
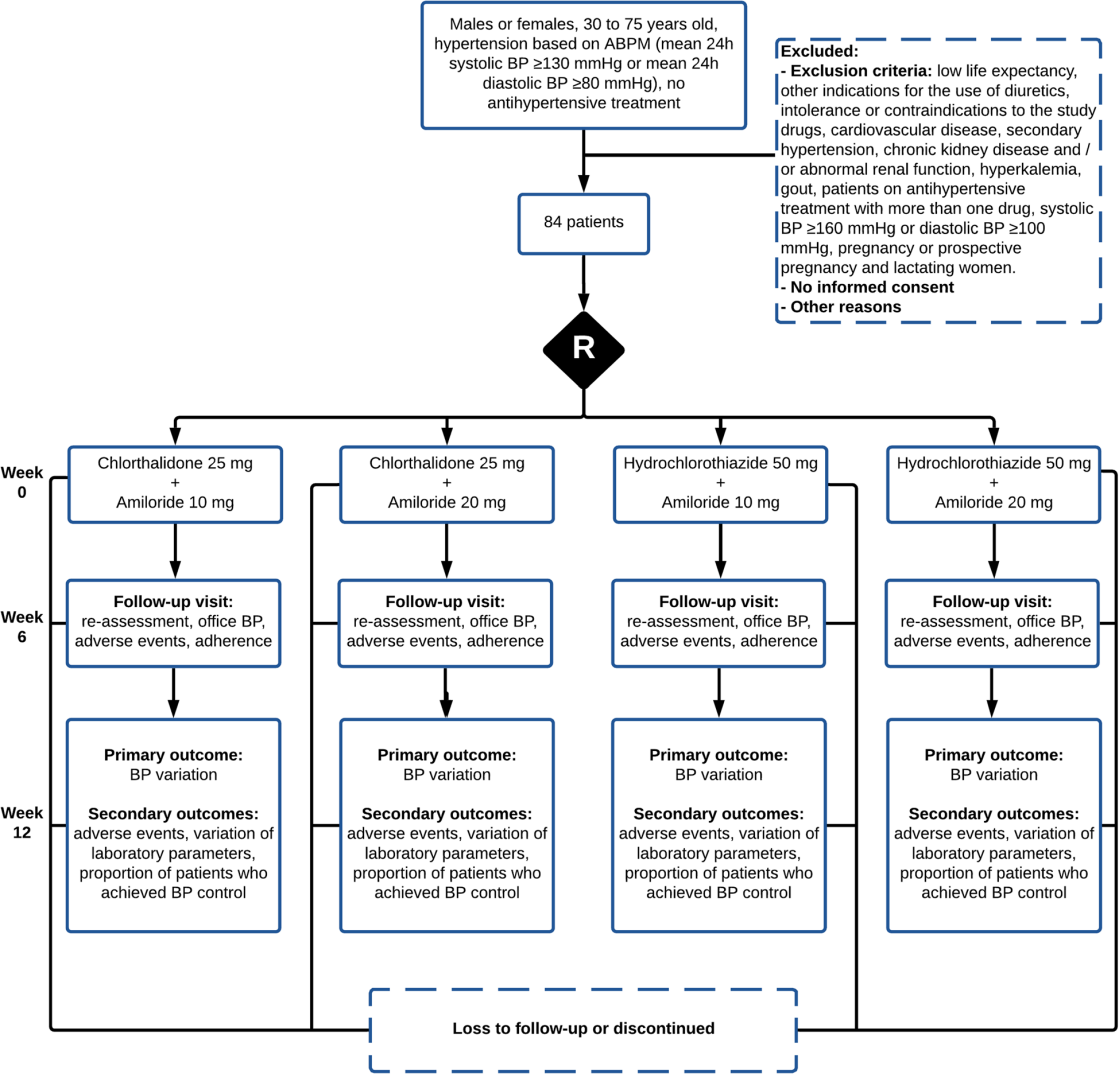
Allocation of participants and timeline. *ABPM* ambulatory blood pressure monitoring, *BP* blood pressure

The schedule of enrollment, interventions, and assessments is presented in Fig. 1. The allocation of participants and timeline are presented in Fig. 2.

### Sample size

For an absolute difference of 6 mmHg in systolic BP on 24-h ABPM, with an alpha of 0.05, power of 80%, and standard deviation of 9 mmHg, it will be necessary to study 76 patients in total. The sample size will be increased by 10% to account for possible losses in follow-up, resulting in 84 patients being randomized (42 for each arm).

### Recruitment

Participants will be recruited from outpatient clinics in HCPA and Instituto de Cardiologia do Rio Grande do Sul, Brazil, and from Basic Health Units (public health system). Patients potentially eligible for the study will be contacted by telephone by the trial investigator, who will explain the study and ascertain the patient’s interest. If interested, the patient will be seen in the Center for Clinical Research of HCPA, where the study consultations will be made. The enrollment period is expected to extend over 24 months.

### Allocation

A computer-generated sequence created by the Random Allocation Software [35] will be used to randomly assign patients to the four interventions, stratified by 24-h systolic BP on ABPM (< 140 or ≥ 140 mmHg), with a 1:1:1:1 allocation using random block sizes to generate equal allocation ratio and parallel groups. The block sizes will not be disclosed, to ensure concealment. The randomization process will be performed before the beginning of the trial, with the random allocation sequence registered in Research Electronic Data Capture (REDCap) [36]. To guarantee concealment of the allocation list, randomization will be implemented through a web-based automated system. All patients who give consent for participation and who fulfill the inclusion criteria will be randomized. An independent researcher not involved in the enrolment will produce the randomization sequence. The research team will be blinded to the randomization sequence and it will be concealed from the researchers enrolling and assessing participants. The interventions will be delivered by researchers with extensive knowledge of the study medication.

### Blinding

The study medication in all groups will have the same color, taste, consistency, odor, and appearance. In this way, patients, care providers, outcome assessors, and the entire research team will be blinded regarding the allocation to treatment groups throughout the study. Unblinding will occur only when knowledge of the actual treatment is absolutely essential for further management of the patient, particularly in the occurrence of serious adverse events.

### Data collection

The research team will be trained to perform anthropometric and BP measurements as well as the application of questionnaires. A laboratory technician will collect blood samples after 12-h fasting. These samples will be forwarded for analysis and discarded shortly after. No biological specimens will be stored for future studies. Study data will be collected and managed using REDCap tools hosted at HCPA.

#### Anthropometric measurements

The following anthropometric measurements will be collected at baseline and close-out visit: body weight, height, waist circumference, and body mass index (BMI). Body weight and height, measured by anthropometric scales, will be used to calculate the body mass index (BMI) using the formula BMI = Weight (kg)/Height squared (m2). BMI values of 18.5 to 24.9 kg/m2 are considered eutrophic values, while individuals with BMI values of 25.0 to 29.9 kg/m2 are overweight and ≥ 30 kg/m2 are obese. The waist circumference, measured at the midpoint between the iliac crest and the lower costal margin, is the most representative anthropometric index of intra-abdominal fat and the simplest reproducible measurement.

#### Office blood pressure

Office blood pressure (OBP) will be measured at all visits with the participant sitting quietly in a chair with feet on the floor, back supported, and arm resting on a desk for > 5 min, according to standardized guidelines [37]. The patient should avoid caffeine, exercise, and smoking for at least 30 min before measurement. The cuff size will be used according to arm circumference. Three measurements will be taken (separated by 1–2 min) with a validated automatic oscillometric BP measuring device (HBP-1100, OMRON Healthcare) and the average recorded. Baseline OBP and final OBP will be considered as the mean of six measurements in two separate visits.

#### 24-h ABPM

ABPM is a method that allows the indirect and intermittent recording of BP for 24-h while patients perform their usual activities during the day. Monitoring requires patients to maintain their normal daily activities with the BP being measured automatically at 15 min intervals (daytime) and 20 min intervals (nighttime) for an entire 24-h period. The systolic BP and diastolic BP will be obtained by ABPM, with the mean values for the 24-h period, daytime, and nighttime being considered for analysis. The normal nocturnal dip will be defined as a drop of > 10% in systolic BP from wakefulness to the period of sleeping. Participants will be evaluated by ABPM at the baseline and at the end of the follow-up. ABPM will be performed using portable monitors (Spacelabs 90,207, Redmond, WA, USA).

#### Laboratory tests

Blood samples will be drawn from all patients at the first and last visits after fasting for 12 h. The following laboratory parameters will be assessed: serum total cholesterol (TC), HDL cholesterol (HDL-C), LDL cholesterol (LDL-C), triglycerides (TG), creatinine, urea, potassium, sodium, magnesium, uric acid, fasting plasma glucose, and hemoglobin A1c (HbA1c). The LDL fraction is calculated in mg/dL, using the Friedewald formula [LDL = TC − HDL − TG/5 (for TG < 400 mg/dL)].

#### Adverse events

Patients will be interviewed at each visit about the occurrence of any adverse events using open questions and a semi-structured questionnaire, including time of onset, duration, and severity; all information will be recorded on an electronic case report form (eCRF). The causal relation to the study drug and the intensity of adverse events will be evaluated by the investigators. Serious adverse events (SAE) must be reported to the institutional review board by the principal investigator within 24 h after the SAE becomes known. Laboratory adverse events, such as hypokalemia, hyperuricemia, and hyperglycemia will be investigated at the final visit.

To improve participant retention, study researchers will make efforts to monitor patients during the study period, including telephone reminders for upcoming visits. Telephone calls will be made to inquire about adverse events if a participant misses a scheduled visit. Furthermore, all participants will be requested to promptly report possible adverse events by telephone. The participant will be advised on the importance of adhering to the treatment protocol not only for validation of the study results, but mainly for their safety and possible health benefits. In order to improve adherence to intervention protocols, we will use pill count to monitor patient compliance. Participants can withdraw from the trial at any time for any reason without their medical care being affected. Data already collected will continue to be used, and the patients will be asked if they are still willing to provide follow-up data. The reason for withdrawal will be documented whenever possible.

### Data collection forms

Study data will be collected and managed using REDCap tools hosted at HCPA. REDCap is a secure, web-based application designed to support data capture for research studies, providing: 1) an intuitive interface for validated data entry; 2) audit trails for tracking data manipulation and export procedures; 3) automated export procedures for seamless data downloads to common statistical packages; and 4) procedures for importing data from external sources. An eCRF will be constructed for data registration. Data integrity will be enforced through valid values and range checks. For data analysis, subject-related data from REDCap will be exported and analyzed in statistics software (IBM SPSS). Before data export, all patient identifiers will be removed.

### Data management

Each participant will be given an identification number at enrolment, and each identification number will have an eCRF. The list over identification codes will be deleted at the end of the study. All trial data, including Trial Master File, eCRF, the source datasheet of the eCRF, and the list of identification codes will be stored in the external server of REDCap with continuous backup. REDCap data are kept for 10 years. Study-related patient documentation and the signed informed consent form will be stored in a patient-specific folder.

### Statistical methods

All data will be analyzed according to the intention-to-treat principle, considering all patients as randomized regardless of whether they received the randomized treatment. Analyses will be performed with the software SPSS for Windows (version 17; SPSS Inc., Chicago, Illinois, USA). A *P* value < 0.05 will be considered statistically significant.

The sample characteristics will be presented by descriptive statistics, and the results will be expressed as mean, standard deviation, and percentage. The comparison of levels of BP among treatment groups at each visit will be done using a *t*-test for independent samples, and a random-effects linear model fitted to systolic and diastolic BP will be used to compare BP by treatment group during follow-up. The random-effects model will include an intercept and a slope to adjust for the within-participant correlation among the longitudinal data with test for interaction. An attempt to measure BP at the end of trial even for participants that abandoned the treatment will be undertaken. The cumulative incidence of adverse events will be analyzed by Chi-square test. Adverse events will be reported with relative risk and 95% confidence intervals. No subgroup or adjusted analyses are planned for this study.

Assuming there will be no interaction between thiazides and amiloride, we intend to carry out pooled analysis of the differences between CTD (CTD 25 mg with amiloride 10 mg and CTD 25 mg with amiloride 20 mg) versus HCTZ (HCTZ 50 mg with amiloride 10 mg and HCTZ 50 mg with amiloride 20 mg) and the differences between amiloride in a lower dose (amiloride 10 mg with CTD 25 mg and amiloride 10 mg with HCTZ 50 mg) versus amiloride in a higher dose (amiloride 20 mg with CTD 25 mg and amiloride 20 mg with HCTZ 50 mg).

### Data monitoring

Due to the short duration of the trial and minimal risks associated with the interventions, a Data Monitoring Committee will not be established, and interim analyses will not be performed. Committees involved in trial coordination and conduct are to be decided elsewhere and will be described in amendments or in the final text.

### Harms

At all follow-up visits, adverse events will be investigated by spontaneous reporting and by a directed questionnaire. An adverse event is considered to be any undesired medical occurrence in a clinical trial participant who has received a pharmaceutical product, even if it does not necessarily have a causal relationship to that treatment. A severe adverse event is considered to be any unfavorable medical occurrence that results in death, threat to life, hospitalization or its prolongation, or persistent or significant disability. The causal relationship to the study drug and the intensity of adverse events will be evaluated by the investigators. The communication of adverse events classified as severe or unexpected will be reported to the Ethics Committee. SAE must be reported by the principal investigator within 24-h after the SAE becomes known. The participant who presents with a severe adverse event will be withdrawn from the study. Withdrawal may also occur in the event of intolerance of the participant to non-severe adverse events. In this case, the procedures for the last visit will be carried out. Laboratory adverse events, such as hypokalemia, hyperuricemia, and hyperglycemia, will be investigated at the final visit. All adverse events and withdrawals due to adverse events will be reported, irrespective of severity, with no frequency threshold.

### Auditing

A quality assurance audit/inspection of this study may be conducted by the competent authority. The quality assurance auditor/inspector will have access to all medical records, the investigator’s study related files and correspondence, and the informed consent documentation that is relevant to this clinical study. The investigator will allow the persons being responsible for the audit or the inspection to have access to the source data/documents and to answer any questions arising. All involved parties will keep the patient data strictly confidential.

### Standard Protocol Items: Recommendations for Interventional Trials checklist

This article followed the Standard Protocol Items: Recommendations for Interventional Trials (SPIRIT) 2013 statement on the writing of a study protocol for a clinical trial [38]. The filled SPIRIT checklist can be found in Additional file 2. The World Health Organization Trial Registration Data Set is provided in Additional file 3.

### Protocol amendments

Any modifications to the protocol which may impact on the conduct of the study or potential benefit to the patient or may affect patient safety, including changes of study objectives, study design, patient population, sample sizes, study procedures, or significant administrative aspects, will require a formal amendment to the protocol. Such amendments will be approved by the Ethics Committee of HCPA prior to implementation. Amendments will be disclosed in the new version of the protocol with reasons.

### Consent

Trained physicians responsible for eligibility will supply informed consent forms to patients willing to participate in the trial. The consent form includes institutional affiliation, the objectives of the study, a description of the testing procedures, explanation about interventions and its randomized allocation nature, information about expected length of time for participation, the potential risks and benefits involved in the study, the costs to the participants, information on anonymized data sharing, and an explanation of the patient’s right to refuse participation or to withdraw consent at any time. A copy of the consent form is given to the participant, and this fact is documented in the subject’s record. The investigator in charge of providing clarification on the study and seeking the participant’s ethical consent must allow the subject sufficient time to decide whether or not to participate in the trial. Once a subject decides to participate, a signed and personally dated informed consent form is obtained from the subject before any trial-related procedure. As there are no plans for use of data from this trial in ancillary studies, no additional consent will be required.

### Confidentiality

Study data will be collected and managed using REDCap tools. All laboratory specimens, reports, data collection, processes, and administrative forms will be identified by a coded identification number to maintain participant confidentiality. After full data analysis, all subject identifiers will be erased. The principal investigator will grant the relevant personnel user rights to view, edit, or overwrite data entries by password as applicable. All edits will be automatically documented in the change history log. Direct access to source data may be granted in the case of monitoring, audit, or inspections. All personnel must treat patient data as confidential. As far as possible, encoded data will be used.

### Access to data

All trial investigators will be given access to the cleaned data sets. Project data sets will be stored in the external server of REDCap hosted at HCPA, and all data sets will be password protected. To ensure confidentiality, data dispersed to project team members will be blinded of any identifying participant information.

### Ancillary and post-trial care

The study drugs have been used for a long time to treat hypertension and are considered safe. The risks of the study are mainly due to the possibility of adverse effects with the drugs. The most frequent adverse effects are anorexia, dyspepsia, dizziness, headache, cramps, and increased urine volume, and usually do not require discontinuation of treatment. In case of adverse effects or problems related to participation in the study that require medical treatment, the investigators will be responsible for the care such that participant do not incur costs.

## Discussion

Thiazide diuretics have good tolerability and proven BP-lowering efficacy, although there is concern about metabolic complications with these agents (e.g., hypokalemia), which can be mitigated by the association of a potassium-sparing diuretic, such as amiloride. This is the first double-blind, randomized controlled trial comparing CTD versus HCTZ combined with amiloride in different doses in relation to BP-lowering efficacy and adverse metabolic effects in patients with primary hypertension. Although the primary outcome (change from baseline in BP) is not a clinical endpoint, BP has been a valid surrogate of the beneficial effects of BP-lowering drugs. Thus, by identifying the diuretic treatment with greater antihypertensive efficacy and lower incidence of adverse effects, this study will provide evidence-based information that could help in the accomplishment of a more effective hypertension treatment.

## Trial status

This is the first version of the protocol (issue date 28 June 2019). The study has not started recruiting participants. We anticipate the study will start by November 2019 and be completed by November 2021.

## Supplementary information

**Additional file 1.** TIDieR checklist.

**Additional file 2.** SPIRIT 2013 checklist: Recommended items to address in a clinical trial protocol and related documents.

**Additional file 3.** World Health Organization Trial Registration Data Set.

## Data Availability

This trial is in accordance with the compliance of the reproducibility standards according to the International Committee of Medical Journal Editors (ICMJE) [39]. Authorship will be determined according to the guidelines of the ICMJE. All trial results, whether positive, negative, or neutral, will be disseminated in peer-reviewed journals, at scientific conferences, and in a public trial data repository. The trial is registered at ClinicalTrials.gov, improving clinical trial transparency and reducing publication bias and selective outcome reporting. We intend to publish the results in an open-access journal, indexed at the Directory of Open Access Journals, with the copyrights transferred to the authors. Also, all materials, raw and treated data, statistical code, and outputs will be publicly shared without restrictions to access the data without an expiration date. Individual participant data will be shared in a de-identified manner, accompanied by a glossary of variables. Only the study guarantor will have the key for re-identification. The repository has not been chosen yet and will be provided in further amendments or in the final report of this study.

## Abbreviations

ABPM: Ambulatory blood pressure monitoring
BMI: Body mass index
BP: Blood pressure
CAPES: Coordenação de Aperfeiçoamento de Pessoal de Nível Superior
CTD: Chlorthalidone
eCRF: Electronic case report form
FIPE: Fundo de Incentivo à Pesquisa e Eventos
HbA1c: Hemoglobin A1c
HCPA: Hospital de Clínicas de Porto Alegre
HCTZ: Hydrochlorothiazide
HDL-C: HDL cholesterol
ICMJE: International Committee of Medical Journal Editors
INCT PREVER: National Institute of Science and Technology for Prevention of Cardiovascular Disease
LDL-C: LDL cholesterol
OBP: Office blood pressure
REDCap: Research Electronic Data Capture
SAE: Serious adverse events
SHEP: Systolic Hypertension in the Elderly Program
SPIRIT: Standard Protocol Items: Recommendations for Interventional Trials
TC: Total cholesterol
TG: Triglycerides
TIDieR: Template for Intervention Description and Replication
