# Supplementary material for: Efficacy of chlorthalidone and hydrochlorothiazide in combination with amiloride in multiple doses on blood pressure in patients with primary hypertension: a protocol for a factorial randomized controlled trial
Source: Trials. 2019 Dec 16;20:736. doi: 10.1186/s13063-019-3909-z (PMC6916111; doi:10.1186/s13063-019-3909-z)
Supplement: Supplementary file 1 — Additional file 1. TIDieR checklist. [file 13063_2019_3909_MOESM1_ESM.docx]

**TIDieR Checklist**

**Efficacy of chlorthalidone and hydrochlorothiazide in combination with amiloride in multiple doses on blood pressure in patients with primary hypertension: description of interventions of a factorial randomized controlled trial.**

1. **Brief name**

Chlorthalidone and hydrochlorothiazide in combination with amiloride in multiple doses.

1. **Why**

Thiazide diuretics, including chlorthalidone and hydrochlorothiazide, have been commonly used as pharmacological agents for the treatment of primary hypertension, with demonstrated blood pressure lowering efficacy at low doses [1-8]. However, there exist concerns regarding adverse metabolic effects such as hypokalemia, hyperglycemia and hyperlipidemia [2, 9, 10], which may be attenuated with the concomitant administration of a potassium-sparing diuretic, such as amiloride [11]. Also, the inclusion of a new diuretic to control adverse effects could offer an additional blood pressure control, especially in higher doses [11, 12]. Amiloride is commonly administered with hydrochlorothiazide, although other fixed-dose combinations of thiazides and potassium-sparing agents are available. It remains unknown whether different diuretics are associated with different clinical outcomes. The amount of blood pressure reduction is the major determinant of reduction in cardiovascular risk in hypertensive patients [13-16], which renders the blood pressure lowering effect among diuretics an appropriate surrogate outcome. This trial aims to investigate the antihypertensive efficacy of chlorthalidone and hydrochlorothiazide, in combination with amiloride in different doses, for the initial management in patients with primary hypertension.

1. **What (materials)**

Participants will receive two simultaneous interventions: a thiazide diuretic (chlorthalidone 25 mg or hydrochlorothiazide 50 mg) and a potassium-sparing diuretic (amiloride 10 mg or amiloride 20 mg). Randomization will be done in 1:1:1:1 ratio, and participants will be randomly assigned to four groups: chlorthalidone 25 mg + amiloride 10 mg, chlorthalidone 25 mg + amiloride 20 mg, hydrochlorothiazide 50 mg + amiloride 10 mg and hydrochlorothiazide 50 mg + amiloride 20 mg. The thiazide diuretic and amiloride will be combined in a single capsule, which will be provided by a compounding pharmacy. The medication will have the same color, taste, consistency, odor and appearance. In this way, patients, researchers, evaluators and the entire research team will be blinded regarding the allocation to the treatment groups throughout the study. The vials containing the capsules of the study drug will be identified only with the study logo, number of capsules dispensed, randomization code and expiry date. The code will be confidentially stored in two independent places, without access by study members.

1. **What (procedures)**

At the time of provision of the medication, the participant will receive a vial containing capsules of the drug and will be advised on the dosage and observation of any symptoms (adverse events) that appear after starting the drug so that it can report on the subsequent consultation. The physician will ensure the understanding of orientations on the administration of the study drug. The participant will be instructed to bring the vial of the medicine in use in the next consultation, even if empty. The patient will also be advised not to take the study medication on the next consultation day, as it should do so during the medical consultation when requested. If the participant attends the consultation, the blood pressure measurement will be performed before the patient takes the study drug. If the patient has forgotten the study drug vial at home, the physician should use one tablet from the next vial to be dispensed to the patient and arrange for delivery of the previously provided vial. The participant will be advised that in case he forgets to take the medicine, he should not take two tablets to compensate for omission and should continue to take normally.

1. **Who provided**

The interventions will be delivered by researchers with extensive knowledge of the study medication and will provide guidance on adherence, dosage, and adverse events to participants at each delivery.

1. **How**

The study medication will be delivered by the researcher to each participant in the scheduled clinical consultation, with sufficient treatment until the next appointment.

1. **Where**

Participants will be recruited from outpatient clinics in Hospital de Clínicas de Porto Alegre and Instituto de Cardiologia do Rio Grande do Sul, Brazil, and from Basic Health Units (public health system) through the review of medical records of patients in the desired age range. Study consultations will take place in the Center for Clinical Research of Hospital de Clínicas de Porto Alegre.

1. **When and how much**

The vials containing capsules of the study drugs will be delivered to patients at randomization (week 0) and intermediate consultation (week 6), with sufficient treatment until the end of the follow up (week 12). Each vial will contain enough capsules for 6 weeks of treatment, when the next appointment will occur. Considering possible variations in the dates of the consultation, depending on holidays or weekends, will be provided 5 additional capsules, totaling 47 capsules in each bottle. Patients will be instructed to take the medication orally in the morning upon waking.

1. **Tailoring**

The medication will be administered as fixed-dose combinations.

1. **Modifications**

Cannot be described until the study is complete.

1. **How well (planned)**

Adherence to trial medication will be assessed by means of pill count. Participants will be instructed to bring the bottles with the remaining capsules in follow up consultations. Since capsule counting will be used as a measure of adherence to treatment, the importance of this will be reinforced even if the vial is empty. The count of capsules will be made by the physician from the bottles brought by the participant. A participant who has used 80% or more of the prescribed drug will be considered a good adherent.

1. **How well (actual)**

Cannot be described until the study is complete.

**References**

1. Materson BJ, Oster JR, Michael UF, Bolton SM, Burton ZC, Stambaugh JE, Morledge J. Dose response to chlorthalidone in patients with mild hypertension. Efficacy of a lower dose. Clin Pharmacol Ther. 1978 Aug;24(2):192-8.
2. Carlsen JE, Køber L, Torp-Pedersen C, Johansen P. Relation between dose of bendrofluazide, antihypertensive effect, and adverse biochemical effects. BMJ. 1990 Apr 14;300(6730):975-8.
3. Materson BJ, Cushman WC, Goldstein G, Reda DJ, Freis ED, Ramirez EA, Talmers FN, White TJ, Nunn S, Chapman RH, et al. Treatment of hypertension in the elderly: I. Blood pressure and clinical changes. Results of a Department of Veterans Affairs Cooperative Study. Hypertension. 1990 Apr;15(4):348-60.
4. Psaty BM, Lumley T, Furberg CD, Schellenbaum G, Pahor M, Alderman MH, Weiss NS. Health outcomes associated with various antihypertensive therapies used as first-line agents: a network meta-analysis. JAMA. 2003 May 21;289(19):2534-44.
5. Harper R, Ennis CN, Sheridan B, Atkinson AB, Johnston GD, Bell PM. Effects of low dose versus conventional dose thiazide diuretic on insulin action in essential hypertension. BMJ. 1994 Jul 23;309(6949):226-30.
6. Freis ED, Thomas JR, Fisher SG, Hamburger R, Borreson RE, Mezey KC, Mukherji B, Neal WW, Perry HM, Taguchi JT. Effects of reduction in drugs or dosage after long-term control of systemic hypertension. Am J Cardiol. 1989 Mar 15;63(11):702-8.
7. Savage PJ, Pressel SL, Curb JD, Schron EB, Applegate WB, Black HR, Cohen J, Davis BR, Frost P, Smith W, Gonzalez N, Guthrie GP, Oberman A, Rutan G, Probstfield JL, Stamler J. Influence of long-term, low-dose, diuretic-based, antihypertensive therapy on glucose, lipid, uric acid, and potassium levels in older men and women with isolated systolic hypertension: The Systolic Hypertension in the Elderly Program. SHEP Cooperative Research Group. Arch Intern Med. 1998 Apr 13;158(7):741-51.
8. Musini VM, Nazer M, Bassett K, Wright JM. Blood pressure-lowering efficacy of monotherapy with thiazide diuretics for primary hypertension. Cochrane Database Syst Rev. 2014 May 29;(5):CD003824.
9. Leung AA, Wright A, Pazo V, Karson A, Bates DW. Risk of thiazide-induced hyponatremia in patients with hypertension. Am J Med. 2011 Nov;124(11):1064-72.
10. Siegel D, Hulley SB, Black DM, Cheitlin MD, Sebastian A, Seeley DG, Hearst N, Fine R. Diuretics, serum and intracellular electrolyte levels, and ventricular arrhythmias in hypertensive men. JAMA. 1992 Feb 26;267(8):1083-9.
11. Brown MJ, Williams B, Morant SV, Webb DJ, Caulfield MJ, Cruickshank JK, Ford I, McInnes G, Sever P, Salsbury J, Mackenzie IS, Padmanabhan S, MacDonald TM; British Hypertension Society's Prevention and Treatment of Hypertension with Algorithm-based Therapy (PATHWAY) Studies Group. Effect of amiloride, or amiloride plus hydrochlorothiazide, versus hydrochlorothiazide on glucose tolerance and blood pressure (PATHWAY-3): a parallel-group, double-blind randomized phase 4 trial. Lancet Diabetes Endocrinol. 2016 Feb;4(2):136-47.
12. Hood SJ, Taylor KP, Ashby MJ, Brown MJ. The spironolactone, amiloride, losartan, and thiazide (SALT) double-blind crossover trial in patients with low-renin hypertension and elevated aldosterone-renin ratio. Circulation. 2007 Jul 17;116(3):268-75.
13. Law MR, Morris JK, Wald NJ. Use of blood pressure lowering drugs in the prevention of cardiovascular disease: meta-analysis of 147 randomized trials in the context of expectations from prospective epidemiological studies. BMJ. 2009 May 19;338:b1665.
14. Blood Pressure Lowering Treatment Trialists' Collaboration, Turnbull F, Neal B, Ninomiya T, Algert C, Arima H, Barzi F, Bulpitt C, Chalmers J, Fagard R, Gleason A, Heritier S, Li N, Perkovic V, Woodward M, MacMahon S. Effects of different regimens to lower blood pressure on major cardiovascular events in older and younger adults: meta-analysis of randomised trials. BMJ. 2008 May 17;336(7653):1121-3.
15. Ettehad D, Emdin CA, Kiran A, Anderson SG, Callender T, Emberson J, Chalmers J, Rodgers A, Rahimi K. Blood pressure lowering for prevention of cardiovascular disease and death: a systematic review and meta-analysis. Lancet. 2016 Mar 5;387(10022):957-967.
16. Thomopoulos C, Parati G, Zanchetti A. Effects of blood pressure-lowering treatment on cardiovascular outcomes and mortality: 14 - effects of different classes of antihypertensive drugs in older and younger patients: overview and meta-analysis. J Hypertens. 2018 Aug;36(8):1637-1647.
