# Supplementary material for: Efficacy of chlorthalidone and hydrochlorothiazide in combination with amiloride in multiple doses on blood pressure in patients with primary hypertension: a protocol for a factorial randomized controlled trial
Source: Trials. 2019 Dec 16;20:736. doi: 10.1186/s13063-019-3909-z (PMC6916111; doi:10.1186/s13063-019-3909-z)
Supplement: Supplementary file 3 — Additional file 3. World Health Organization Trial Registration Data Set. [file 13063_2019_3909_MOESM3_ESM.docx]

**World Health Organization Trial Registration Data Set**

| **Data category** | **Information** |
| --- | --- |
| Primary Registry and Trial Identifying Number | ClinicalTrials.gov NCT03928145 |
| Date of Registration in Primary Registry | April, 2019 |
| Secondary Identifying Numbers | Not applicable |
| Source(s) of Monetary or Material Support | Fundo de Incentivo à Pesquisa e Eventos (FIPE) and National Institute of Science and Technology for Prevention of Cardiovascular Disease (INCT PREVER). |
| Primary Sponsor | Hospital de Clínicas de Porto Alegre, Universidade Federal do Rio Grande do Sul |
| Secondary Sponsor(s) | Not applicable |
| Contact for Public Queries | Principal Investigator: Flávio Danni Fuchs, MD, PhD  Address: Division of Cardiology, Hospital de Clínicas de Porto Alegre, R. Ramiro Barcellos 2350, Porto Alegre/RS, ZIP 90035-903, Brazil  Telephone: +55 51 3359.8344  Email: ffuchs@hcpa.edu.br  Affiliation: Hospital de Clínicas de Porto Alegre, Universidade Federal do Rio Grande do Sul |
| Contact for Scientific Queries | Principal Investigator: Flávio Danni Fuchs, MD, PhD  Address: Division of Cardiology, Hospital de Clínicas de Porto Alegre, R. Ramiro Barcellos 2350, Porto Alegre/RS, ZIP 90035-903, Brazil  Telephone: +55 51 3359.8344  Email: ffuchs@hcpa.edu.br  Affiliation: Hospital de Clínicas de Porto Alegre, Universidade Federal do Rio Grande do Sul  Guarantor of the trial: Flávio Danni Fuchs, MD, PhD  Address: Division of Cardiology, Hospital de Clínicas de Porto Alegre, R. Ramiro Barcellos 2350, Porto Alegre/RS, ZIP 90035-903, Brazil  Telephone: +55 51 3359.8344  Email: ffuchs@hcpa.edu.br  Affiliation: Hospital de Clínicas de Porto Alegre, Universidade Federal do Rio Grande do Sul |
| Public Title | Efficacy of chlorthalidone and hydrochlorothiazide in combination with amiloride in multiple doses on blood pressure in patients with primary hypertension. |
| Scientific Title | Efficacy of chlorthalidone and hydrochlorothiazide in combination with amiloride in multiple doses on blood pressure in patients with primary hypertension: a factorial randomized controlled trial. |
| Countries of Recruitment | Brazil |
| Health Condition(s) or Problem(s) Studied | Hypertension, antihypertensive treatment |
| Intervention(s) | Active intervention: chlorthalidone 25 mg + amiloride 20 mg (one capsule per day for 12 weeks)  Active intervention: chlorthalidone 25 mg + amiloride 10 mg (one capsule per day for 12 weeks)  Active intervention: hydrochlorothiazide 50 mg + amiloride 20 mg (one capsule per day for 12 weeks)  Active control: hydrochlorothiazide 50 mg + amiloride 10 mg (one capsule per day for 12 weeks) |
| Key Inclusion and Exclusion Criteria | Inclusion criteria: adults (age 30 to 75 years) with diagnosis of primary hypertension based on ABPM (mean 24-h systolic BP ≥130 mmHg or mean 24-h diastolic BP ≥80 mmHg) and without current use of antihypertensive medication.  Exclusion criteria: low life expectancy, other indications for the use of diuretics, intolerance or contraindications to the study drugs, cardiovascular disease (heart failure, myocardial infarction or stroke), secondary hypertension, chronic kidney disease and / or abnormal renal function (creatinine >1.5 mg/dL), hyperkalemia (serum potassium >5.5 mEq/L) or gout. Patients on antihypertensive treatment with more than one drug, with systolic BP ≥160 mmHg or diastolic BP ≥100 mmHg measured through office BP, with pregnancy or prospective pregnancy during the study and lactating women will also be excluded. |
| Study Type | Interventional  Allocation: randomized  Allocation concealment mechanism: randomization will be implemented through a web-based automated system  Sequence generation: computer generated sequence created by the Random Allocation Software  Masking: double blind (patients, researchers, evaluators and the entire research team will be blinded regarding the allocation to the treatment groups)  Assignment: factorial  Primary purpose: treatment  Phase III |
| Anticipated date of First Enrollment | November 2019 |
| Sample Size | Planned: 84  Enrolled: 0 |
| Recruitment Status | Pending |
| Primary Outcome(s) | 1 - Difference between the treatment arms in mean change from baseline in 24-h systolic blood pressure measured by ambulatory blood pressure monitoring at 12 weeks.  2 - Difference between the treatment arms in mean change from baseline in 24-h diastolic blood pressure measured by ambulatory blood pressure monitoring at 12 weeks. |
| Key Secondary Outcomes | 1 - Difference between the treatment arms in mean change from baseline in daytime and nighttime systolic and diastolic blood pressure measured by ambulatory blood pressure monitoring at 12 weeks.  2 - Difference between the treatment arms in mean change from baseline in systolic and diastolic blood pressure measured by office blood pressure at 12 weeks.  3 - Difference between treatment arms in the proportion of participants reporting adverse events in the 12 weeks following randomization.  4 - Difference between the treatment arms in mean change from baseline in laboratory parameters measured at 12 weeks.  5 - Difference between treatment arms in the proportion of participants achieving BP control at 12 weeks. |
| Ethics Review | 1 - Status: approved  2 - Date of approval: April 2019  3 - Name and contact details of Ethics committee: Ethics Committee of Hospital de Clínicas de Porto Alegre. R. Ramiro Barcellos 2350, Porto Alegre/RS, Brazil. Telephone: +55 51 3359.7640. Email: cep@hcpa.edu.br. |
| Completion date | Trial not completed. |
| Summary Results | Not applicable. |
| IPD sharing statement | Plan to share IPD: yes  Plan description: all materials, raw and treated data, statistical code and outputs will be publicly shared without restrictions to access the data neither expiration date. The repository was not chosen yet and will be provided in further amendments or in the final report of this study. |
